# Evaluating the Clinical Impact of BioFire Spotfire R/ST on the Management of Pediatric Respiratory Presentations in the Emergency Department: A Pre–Post Cross-Sectional Study in Chile

**DOI:** 10.3390/v18010139

**Published:** 2026-01-22

**Authors:** Dona Benadof, Mirta Acuña, Yennybeth Leiva, Daniel Conei

**Affiliations:** 1 Laboratorio Clínico, Hospital Roberto del Rio, Santiago 8380457, Chile; 2 Departamento de Pediatría y Cirugía, Facultad de Medicina, Universidad de Chile, Santiago 8380456, Chile; mirta.i.acuna@gmail.com (M.A.); yennybeth@gmail.com (Y.L.); 3 Centro de Investigación Clínica y Avanzada, Facultad de Medicina, Universidad de Chile, Santiago 8380456, Chile; 4 Unidad de Infectología, Hospital Roberto del Rio, Santiago 8380457, Chile; 5 Departamento de Procesos Terapéuticos, Facultad de Ciencias de la Salud, Universidad Católica de Temuco, Temuco 4780000, Chile; dconei@uct.cl

**Keywords:** SpotFire, multiplex PCR panels, respiratory viruses, emergency room

## Abstract

Respiratory infections represent one of the leading causes of pediatric consultations and hospitalizations in Chile, where rapid etiological identification is essential for clinical decision-making. We evaluated the impact of implementing the BIOFIRE^®^ SPOTFIRE^®^ Respiratory (R) Panel in the pediatric Emergency Department of a public referral hospital in Santiago, using a pre–post cross-sectional design comparing two winter periods (July 2023 vs. July 2024). Clinical records, laboratory data, and operational indicators were analyzed to assess changes in diagnostic yield, turnaround time, hospitalizations, discharges, supplementary test requests, and antimicrobial use. A total of 470 patients were included (224 in 2023; 246 in 2024). The etiological detection rate increased from 58.0% to 87.8% after the implementation of Spotfire^®^ (*p* < 0.0001), with marked increases in the identification of adenovirus, RSV, rhinovirus/enterovirus, and seasonal coronaviruses. Rapid molecular testing was associated with a significant rise in emergency department discharges (23.7% vs. 57.3%; *p* < 0.0001) and a reduction in hospitalizations (76.3% vs. 42.7%; *p* < 0.0001) and readmissions (9.2% vs. 0.5%; *p* < 0.0001). Requests for complete blood counts, chest X-rays, and antimicrobial prescriptions at discharge also decreased significantly. These effects persisted in key subgroups, including infants and children with comorbidities. In this high-demand winter setting, the BIOFIRE^®^ SPOTFIRE^®^ R Panel improved diagnostic performance and supported more efficient and targeted clinical management.

## 1. Introduction

Respiratory infections are a leading cause of death in children and have also been associated with an increased burden of disease worldwide, with a greater impact on low- and middle-income countries [1,2]. The COVID-19 pandemic highlighted the high socioeconomic cost and global impact of respiratory viruses when they cause widespread infection [2,3,4]. In pediatrics, the etiology of respiratory infections is predominantly viral, and these are one of the main causes of hospitalization in children under 5 years of age, especially in low- and middle-income countries [4]. Mazur et al. reported that globally, 33 million respiratory infections associated with respiratory syncytial virus (RSV) occur annually in children under 5 years of age, with 3.6 million hospitalizations and approximately 118,200 deaths [5]. In Chile, in 2023, 55.8% of pediatric outpatient visits to emergency services were for respiratory tract infections (RTIs). Among the most frequently diagnosed viruses are RSV, influenza, parainfluenza, human adenovirus (HAdV), and metapneumovirus [6,7,8].

The conventional diagnosis of respiratory infections in pediatric emergency departments in Latin America was mainly based on direct fluorescence assay (DFA), lateral flow immunochromatography, and, occasionally, molecular biology assays for multiple pathogens, which typically include 2 to 4 pathogens (RSV, influenza, SARS-CoV-2 [8,9,10]). However, recent advances in molecular biology have revolutionized the diagnosis of infectious diseases, changing the paradigm of virological diagnosis. Initially, this shift was from culture-based methods, known as the “gold standard” for viral detection, to diagnosis through rapid nucleic acid amplification tests (polymerase chain reaction or PCR). Currently, there are commercially available assays capable of detecting more than 20 respiratory pathogens in 45 to 90 min that can be used in healthcare institutions, both in emergency departments and other areas, including outpatient settings [11].

Rapid and accurate identification of the causative agent is essential to guide optimal patient management and the timely implementation of infection control measures, isolation in emergency departments, referral to critical care services such as the ICU, or enabling home discharge with specific treatments [12,13].

In this scenario, the BIOFIRE SPOTFIRE R Panel represents a significant innovation compared to other platforms available in the Latin American market.

Approved by the FDA under 510(k) and with a CLIA waiver, this test allows the simultaneous detection of multiple respiratory pathogens in approximately 17 min, a turnaround time up to three times faster than its predecessor, BioFire^®^ FilmArray^®^ RP2.1plus. Furthermore, it integrates nucleic acid extraction, reverse transcription, nested amplification, fluorescence detection, and automated melting curve analysis into a closed system, simplifying workflow with a single step for the operator, reducing the risk of contamination [13].

However, the BIOFIRE SPOTFIRE R Panel lacks published clinical trials in Latin America demonstrating its value. Our study aims to evaluate the impact of Spotfire^®^ on clinical outcomes in a pediatric emergency department in Chile, specifically regarding antimicrobial use, requests for additional tests, response time, hospitalizations, and operational actions in patient care.

## 2. Material y Methods

### 2.1. Study Design

An analytical interventional study, using a pre- and post-intervention methodology, was conducted on the clinical use of the Spotfire panel. A cross-sectional design was implemented in the period before the intervention (2023) and the period after (2024) in a pediatric emergency unit of a public hospital in Santiago, Chile.

### 2.2. Population

The study was conducted in the Emergency Department of Roberto del Río Children’s Hospital (HRRIO), the sole public pediatric referral center within the North Metropolitan Health Service, located in Santiago, Chile. HRRIO is a high-complexity institution that provides care to an estimated pediatric population of approximately 200,000 children under 15 years of age, according to projections from the 2024 Chilean National Census. The Emergency Department operates continuously, 24 h per day, 7 days per week. For this study, all consultations registered during the first three weeks of July (21 July) in both evaluated years were included.

### 2.3. Inclusion and Exclusion Criteria

Inclusion criteria were pediatric patients (<15 years) presenting to the Emergency Department who met at least one of the following conditions: (1) axillary fever ≥38 °C with respiratory symptoms (cough, coryza, or odynophagia); (2) re-consultation for a presumed viral febrile respiratory illness; (3) infants <3 months with fever of unknown origin in good general condition and fulfilling all Rochester criteria; (4) suspected adenovirus infection, defined as fever ≥38 °C plus odynophagia, tonsillar exudate, conjunctivitis, or confirmed epidemiological contact; or (5) suspected influenza infection, defined as fever ≥38 °C plus headache, odynophagia, myalgia, ocular pain, or confirmed epidemiological contact. These criteria were based on pre-existing emergency department guidelines.

Exclusion criteria were clinical shock, impaired level of consciousness, respiratory failure requiring immediate stabilization, or inadequate/insufficient respiratory sample.

### 2.4. Laboratory Analysis

Nasopharyngeal swab samples collected during the 2024 period were analyzed using the BIOFIRE SPOTFIRE R Panel (BioMérieux, Salt Lake City, UT, USA) between July and August 2024. Samples were left at room temperature prior to analysis. The BIOFIRE SPOTFIRE R Panel cartridge is a closed, disposable system containing all the reagents necessary for sample preparation, reverse transcription, polymerase chain reaction, and detection to isolate, amplify, and detect nucleic acid from various respiratory pathogens in a nasopharyngeal swab (NPS) or throat swab (TS) sample. Reagents and equipment were handled according to the reagent insert and equipment manual. The detection consisted of the following viral and bacterial analyses: adenovirus, SARS-CoV-2 coronavirus, coronavirus (seasonal), human metapneumovirus, human rhinovirus/enterovirus, influenza A A/H1-2009 virus, influenza A A/H3 virus, influenza A virus, influenza B virus, parainfluenza virus, respiratory syncytial virus, *Bordetella parapertussis*, *Bordetella pertussis*, *Chlamydia pneumoniae*, *Mycoplasma pneumoniae*, and *Mycoplasma hominis* [13]. During 2023, the standard of care for virological diagnosis was by DFA, with a sample obtained from a nasopharyngeal swab using a kit SimulFluor^®^ Respiratory Virus (Light Diagnostics Merck Millipore, Burlington, MA, USA) with detection of syncytial respiratory virus, metapneumovirus, adenovirus, influenza A and B, and parainfluenza virus.

### 2.5. Definitions

The Chilean public health system uses the Emergency Severity Index (ESI) triage system in Emergency departments. This score categorizes patients from I to V, where I is the patient who requires immediate stabilization due to life-threatening risk and V is the one who does not require any clinical resources to be stable.

ESI: 1,2,3,4 [14].

### 2.6. Data Collection and Handling

Study data were collected and managed using REDCap electronic data capture tools hosted in Facultad de Medicina de la Universidad de Chile. 1 2 REDCap (Research Electronic Data Capture) is a secure, web-based software platform designed to support data capture for research studies, providing: (1) an intuitive interface for validated data capture; (2) audit trails for tracking data manipulation and export procedures; (3) automated export procedures for seamless data downloads to common statistical packages; and (4) procedures for data integration and interoperability with external sources. Patient data were obtained from the emergency unit’s clinical record, the clinical laboratory’s LIS, and the electronic clinical record in case of hospitalization.

### 2.7. Statistical Analysis

For the sample size estimation, a statistical power of 80%, a statistical significance level of 5%, and an effect size of 0.24 were considered. Data from the Emergency Department of the Roberto del Río Hospital were used, where a total of 80,308 visits were recorded in 2023, of which 18,950 (23.6%) were for respiratory causes in pediatric patients. In 2024, 81,503 emergency visits were recorded, with 17,349 (21.3%) for respiratory causes. Based on this data and considering the use of contingency tables with 3 degrees of freedom and a 10% loss to follow-up, a total sample size of 200 subjects was estimated for both pre- and post-intervention. All these parameters were subjected to the statistical software GPower 3.1, considering the use of contingency tables and the statistical tests to be used.

An exploratory analysis of the data was performed for the purpose of clinical recategorization. Subsequently, a descriptive analysis of the general and clinical characteristics of the study population was conducted, expressing quantitative variables as mean ± standard deviation and qualitative variables as absolute frequencies and percentages (%). Given the sample size, the Kolmogorov–Smirnov test was used to assess normality. For comparison of categorical variables, the chi-square test or Fisher’s exact test was used. For quantitative variables, Student’s *t*-test or the Mann–Whitney U test was used, depending on the normality of the data [15]. Regarding the comparison of outcomes between the two groups, the study population was divided into two age groups: older than 9 months and younger than 9 months. This was done to avoid the confounding variable of immunization with monoclonal antibodies against respiratory syncytial virus (RSV), which was also implemented during 2024 and was indicated for all infants born from 1 October 2023, onward.

A *p*-value < 0.05 was considered statistically significant for all analyses described. The statistical program used was GraphPad Prism 10.6.0 (Boston, MA, USA).

### 2.8. Ethical Aspects

The study was approved by the Hospital Director and the Scientific Ethics Committee of the North Metropolitan Health Service.

## 3. Results

224 patients were included in 2023 and 246 patients in 2024; there was no statistical difference in the age distribution between both periods. (Table 1) The main reasons for consultation in both periods were fever, cough, and coryza, with no statistical differences.

In terms of the etiological identification performance of diagnostic techniques, this increased from 58.0% in 2023 to 87.8% in 2024 (*p* < 0.0001). In 2024, a higher proportion of adenoviruses were identified (8.1% vs. 2.2%, *p* < 0.0001), along with a significant increase in respiratory syncytial virus (41.9% vs. 29.0%; *p* = 0.0037) and the emergence of seasonal coronaviruses (5.7%) and rhinovirus/enteroviruses (36.6%), which had not been detected the previous year. Negative results decreased markedly in 2024 (12.2% vs. 42.0%; *p* < 0.001). During the 2024 period, viral co-detection was identified in 90 cases, including 8 cases with the simultaneous detection of three distinct viruses. These cases represented 34.28% of hospitalizations during the period, with a stay of 5.03 + 3.48 days, and admission to ICU occurred in 57.14% of the total cases in 2024, with 4.87 + 2.90 days. Human metapneumovirus remained a prevalent agent in both periods, with no significant differences (23.2% vs. 18.7%). Parainfluenza virus circulation also increased in 2024 (5.7% vs. 3.1%), as shown in Table 2. In terms of undiagnosed viral etiology in 2023, it is noteworthy that in the case of rhinovirus, 34% of patients were hospitalized, and of these, 23% had comorbidities.

In 2024, the proportion of patients with positive test results increased markedly (58.0% vs. 87.8%; *p* < 0.001), while hospitalizations and readmissions decreased substantially (83.1% vs. 44.0% and 9.2% vs. 0.5%, respectively; both *p* < 0.001). Comparing the period before the intervention (2023), when direct immunofluorescence (DFA) was used, with the period after the implementation of BIOFIRE SPOTFIRE R Panel, significant changes in clinical outcomes were observed. The proportion of discharges from the emergency department increased from 23.7% to 57.3% (*p* < 0.0001), while hospitalizations decreased from 76.3% to 42.7% (*p* < 0.0001). A similar trend was observed among infants under one year of age, with an increase in discharges from 9.4% to 30.5% (*p* < 0.0001) and a decrease in hospitalizations from 45.1% to 22.8% (*p* < 0.0001). In patients with comorbidities, discharges also increased significantly (4.9% vs. 14.2%; *p* = 0.0007), although no significant differences were found in hospitalization rates between the two periods (21.9% vs. 17.5%; *p* = 0.2303). Regarding length of stay in the emergency department, the results were mixed. The overall average was similar between the two periods (3.24 ± 2.05 vs. 3.07 ± 2.35 h; *p* = 0.6142), and no differences were observed in children under one year of age (3.28 ± 2.23 vs. 3.33 ± 2.83 h; *p* = 0.9436). However, the average stay increased among hospitalized patients (3.31 ± 2.16 vs. 3.95 ± 2.01 h; *p* = 0.0132), as well as among hospitalized infants (2.78 ± 2.03 vs. 3.87 ± 2.35 h; *p* = 0.0043). No significant differences were detected in the subgroup with comorbidities (Table 3).

Regarding the request for additional tests, the request for complete blood counts decreased significantly from 122/224 (54.46%) in 2023 to 91/246 (36.99%) in 2024 (*p* < 0.0001); the same was true for chest X-rays, from 184/200 (92.00%) to 163/200 (81.5%) (*p* = 0.0032), and the prescription of antimicrobials at discharge decreased from 59 (26.34%) in 2023 to 42 (17.07%) in 2024 (*p* = 0.0146). Meanwhile, the use of oseltamivir showed no significant differences (3 [1.34%] vs. 2 [0.81%]; *p* = 0.5781).

As shown in Table 4, in the subgroup of infants older than 9 months of age, who in 2024 were not the target population for immunization with nirsevimab, hospitalizations overall decreased from 69.84% to 43.57% (*p* < 0.001), with a significant decrease in the number of days of hospitalization, along with a decrease in hospitalizations in ICU from 15.08% to 6.25%.

## 4. Discussion

The implementation of this type of rapid diagnostic test was associated with significant improvements in both etiological diagnosis and clinical outcomes. The increase in the viral identification rate, from 58.0% in 2023 to 87.8% in 2024, reflects greater diagnostic sensitivity compared to the previous technique (DFA), especially in detecting pathogens such as adenovirus, respiratory syncytial virus (RSV), seasonal coronaviruses, and rhinovirus/enterovirus, whose circulation had not been recorded the previous year.

The reduction in turnaround time since the introduction of the BIOFIRE SPOTFIRE R Panel was substantial, from 10 h in 2023 to just over 1 h in 2024. This can clearly impact the length of stay in emergency units, optimizing resource management. Meltzer et al. concluded that this molecular test was associated with a reduction in antibiotic prescriptions for viral infections, shorter stays in the emergency department, high clinician confidence, and high patient satisfaction [16]. These data support the role of molecular biology as a point-of-care test, enabling early management in the emergency room. Previously, Hu et al. had described something similar with multi-pathogen molecular technologies [2]. Despite the significant reduction in diagnostic turnaround time following the implementation of BIOFIRE^®^ SPOTFIRE^®^ R, a modest but statistically significant increase in emergency department length of stay was observed among patients who were ultimately hospitalized. This finding likely reflects factors external to the microbiological diagnosis, such as the high occupancy of pediatric beds during the winter season of 2024, which resulted in prolonged waiting times for transfer to inpatient wards or critical care units. Also, the early availability of a viral diagnosis enabled a more comprehensive clinical assessment and prolonged stabilization prior to hospitalization, particularly in infants, which may have contributed to this increase without adversely affecting overall emergency department flow.

From a clinical perspective, the observed changes in hospitalization rates, emergency department discharges, and readmissions suggest a direct impact of rapid and accurate diagnosis on medical decision-making. The reduction in hospitalizations (76.3% vs. 42.7%) and readmissions (9.2% vs. 0.5%), along with the increase in emergency department discharges (23.7% vs. 57.3%), suggests greater confidence among the medical team in outpatient management, likely supported by the immediate availability of more comprehensive diagnostic results. This trend was consistent in subgroups such as infants under one year of age and patients with comorbidities, although a significant reduction in hospitalizations was not observed in the latter group, which may be related to greater underlying clinical complexity.

Furthermore, a significant decrease was observed in the number of requests for additional tests (blood counts and chest X-rays), suggesting a more rational use of diagnostic resources and a reduction in antimicrobial prescriptions at discharge, which could be linked to better differentiation between viral and bacterial infections. This reduction in unnecessary antibiotic use is especially relevant in the context of antimicrobial resistance control policies and diagnostic stewardship. Similarly, Walls et al. demonstrated that the use of PCR in the Emergency Department (ED) could decrease antimicrobial prescriptions, improving the confidence of the medical team and reducing patient wait times [17]. The results presented here complement those reported in the medical literature. Syndromic panels in the ED, such as the BIOFIRE SPOTFIRE R Panel, can directly impact hospitalization rates, antimicrobial prescriptions, and the use of complementary diagnostic techniques in the ED. The literature is controversial regarding clinical outcomes according to Vos et al. [18].

Regarding the length of stay in the emergency department, while the overall average remained unchanged, it increased in patients with the indication of hospitalization patients was observed, possibly related to waiting for beds or more extensive clinical evaluation after a viral diagnosis was confirmed or discarded. It should also be noted that patients with a quick diagnosis and good clinical condition were sent home, and the select group of hospitalized patients remained on standby awaiting bed availability. In any case, this increase was modest and does not appear to have negatively impacted the overall flow of the service.

In patients with comorbidities, although rapid molecular testing contributed to increased diagnostic certainty and supported clinical decision-making, the underlying clinical complexity remained a central determinant of hospitalization. In this subgroup, virological information appears to play a complementary rather than a decisive role, which is consistent with routine clinical practice in higher-risk pediatric patients. Nevertheless, knowledge of the specific microbiological etiology improves parental and caregiver satisfaction, institutional epidemiological surveillance, outbreak recognition, and the optimization of follow-up monitoring and treatment strategies.

The marked reduction observed in hospitalization rates cannot be attributed solely to earlier diagnosis but rather to improved etiological accuracy. Rapid and reliable identification of respiratory viruses enabled a more confident distinction between self-limited viral infections and conditions requiring inpatient care, thereby increasing clinician confidence in outpatient management and supporting more efficient decision-making in the emergency department.

Since the use of nirsevimab was implemented universally in infants under 9 months of age at the time of the intervention (July 2024), it was decided to evaluate the group older than 9 months, so that the results would not be influenced by the variable “use of nirsevimab”, to observe if there was a change in the virological identification, in the complementary tests requested, the percentage of hospitalizations and the cause of hospitalizations. In this subgroup of analysis, a decrease in hospitalization, number of days of hospitalization, and admission to the ICU was demonstrated. Eventually, it could be shown that the intervention effectively had an impact without the bias of nirsevimab use [19].

Viral co-detection was a frequent finding following the implementation of multiplex PCR, which is expected when using higher-sensitivity diagnostic techniques. In our cohort, cases with viral co-detection did not necessarily exhibit worse clinical outcomes compared with single-virus infections, reinforcing the importance of interpreting these results within the clinical context rather than automatically assuming increased disease severity associated with the presence of multiple pathogens. A complicating factor therein is that identification of a viral pathogen from a respiratory tract sample may not necessarily attribute causation therefore does not necessarily imply greater severity [20,21].

### Limitations

This study reflects the reality of a public pediatric hospital in Chile, and for that reason, the results may not be fully applicable to other clinical settings or healthcare systems. The point-of-care molecular platform was implemented within the emergency laboratory rather than inside the emergency department itself, which may influence how the findings are interpreted in terms of workflow and operational dynamics. Patient selection depended on a diagnostic algorithm used to determine eligibility for molecular testing, creating the possibility of selection bias that should be considered when interpreting the results.

Given the nature of the study, a cost-effectiveness analysis of the diagnostic strategy was not possible, which limits the assessment of its economic impact and potential scalability. However, it must be acknowledged that the price difference between DFA and multiplex PCR assays is a critical factor for stakeholders in public health systems, and therefore, future studies are needed to evaluate the economic impact, scalability, and sustainability of this technology. Additionally, the study covered only a three-week period during the winter season, traditionally the time of highest respiratory demand. This narrow time window makes the observations vulnerable to year-to-year epidemiological fluctuations that could modify the circulation patterns of respiratory viruses.

## 5. Conclusions

In this real-world evaluation conducted in a public pediatric hospital, the BIOFIRE SPOTFIRE R Panel improved significatively the diagnostic certainty and supported safer outpatient management, reducing hospital pressure during the period of highest respiratory demand. Beyond its clinical benefits, the intervention contributed to a more rational use of healthcare resources, aligning with national priorities in antimicrobial stewardship and optimization of emergency services. While limited in scope and duration, the study provides compelling evidence that rapid syndromic testing may play a strategic role in strengthening pediatric respiratory care pathways in Latin America.

## Figures and Tables

**Table 1:** General and clinical characteristics of the patients studied.

	2023	2024	*p* Value
*Sample size (n)*	224	246	-
*Age (mean months ± SD)*	27.55 ± 21.92	23.58 ± 20.35	0.5093 ^b^
*Age categorized, n (%)*			0.9154 ^a^
* Newborn*	20 (8.93%)	18 (7.32%)	
* Infant*	102 (45.54%)	114 (46.34%)	
* Toddler*	39 (17.41%)	50 (20.32%)	
* Preschooler*	47 (20.98%)	49 (19.92%)	
* School-age child*	14 (6.25%)	14 (5.69%)	
* Adolescent*	2 (0.89%)	1 (0.41%)	
*Gender, n (%)*			0.1435 ^a^
* Male*	117 (52.23%)	145 (58.94%)	
* Female*	107 (47.77%)	101 (41.06%)	
*Medical history*			0.1759 ^a^
* Congenital heart disease*	11 (4.91%)	4 (1.63%)	
* Asthma, unspecified*	17 (7.59%)	12 (4.88%)	
* Bronchopulmonary dysplasia*	4 (1.79%)	4 (1.63%)	
* Atopic dermatitis*	1 (0.45%)	2 (0.81%)	
* Allergic rhinitis*	2 (0.89%)	3 (1.22%)	
* Prematurity*	5 (2.23%)	4 (1.63%)	
* Obesity*	15 (6.7%)	30 (12.2%)	
* Other*	21 (9.38%)	29 (11.79%)	

^a^ Chi-square. ^b^ Student’s *t*-test.

**Table 2:** Etiology according to viral result.

	2023	2024	*p* Value
*Positive, n (%)*	130 (58.04%)	216 (87.80%)	**<0.0001 ^a^**
*Adenovirus, n (%)*	5 (2.23%)	20 (8.13%)	**0.0044 ^a^**
*Coronavirus (seasonal), n (%)*	-	14 (5.96%)	-
*Human metapneumovirus, n (%)*	52 (23.21%)	46 (18.70%)	0.2288 ^a^
*Human rhinovirus/enterovirus, n (%)*	-	90 (36.59%)	-
*Influenza A virus, n (%)*	-	2 (0.81%)	-
*Influenza B virus, n (%)*	1 (0.45%)	-	-
*Parainfluenza virus, n (%)*	7 (3.13%)	14 (5.69%)	0.1787 ^a^
*Respiratory syncytial respiratory, n (%)*	65 (29.02%)	103 (41.87%)	**0.0037 ^a^**
*Chlamydia pneumoniae, n (%)*	-	2 (0.81%)	-
*Mycoplasma pneumoniae, n (%)*	-	1 (0.41%)	-
*Negative, n (%)*	94 (41.96%)	30 (12.20%)	**<0.0001 ^a^**

^a^ Chi-square. Bold text highlights *p* value < 0.05.

**Table 3:** Clinical resolution times in the pediatric emergency department (ED).

	2023	2024	*p* Value
*Discharge to home, n (%)*	53 (23.66%)	141 (57.32%)	**<0.0001 ^a^**
* Length in ED (mean hours ± SD)*	3.24 ± 2.05	3.07 ± 2.35	0.6142 ^b^
*Hospitalization, n (%)*	171 (76.34%)	105 (42.68%)	**<0.0001 ^a^**
* Length in ED (mean hours ± SD)*	3.31 ± 2.16	3.95 ± 2.01	**0.0132 ^b^**
*Discharge to home < 1 year, n (%)*	21 (9.38%)	75 (30.49%)	**<0.0001 ^a^**
* Length in ED (mean hours ± SD)*	3.28 ± 2.23	3.33 ± 2.83	0.9436 ^c^
*Hospitalization < 1 year, n (%)*	101 (45.09%)	56 (22.76%)	**<0.0001 ^a^**
* Length in ED (mean hours ± SD)*	2.78 ± 2.03	3.87 ± 2.35	**0.0043 ^b^**
*Discharge to home with comorbidity, n (%)*	11 (4.91%)	35 (14.23%)	**0.0007 ^a^**
* Length in ED (mean hours ± SD)*	4.00 ± 1.61	2.94 ± 2.07	0.1289 ^c^
*Hospitalization with comorbidity, n (%)*	49 (21.88%)	43 (17.48%)	0.2303 ^a^
* Length in ED (mean hours ± SD)*	3.59 ± 2.24	4.00 ± 2.00	0.3625 ^c^

^a^ Chi-square. ^b^ Student’s *t*-test. ^c^ Mann–Whitney’s U, Bold text highlights *p* value < 0.05.

**Table 4:** Turnaround time and clinical characteristics in patients < 9 months.

	2023	2024	*p* Value
*Turnaround time, (mean hours ± SD)*	11.59 ± 7.61	1.05 ± 0.60	**<0.0001 ^b^**
*Cases < 9 meses, n (%)*	98 (43.75%)	102 (41.46%)	0.6165 ^a^
*Gender*			0.8136 ^a^
*Male, n (%)*	57 (58.16%)	61 (59.80%)	
*Female, n (%)*	41 (41.84%)	41 (40.20%)	
*Comorbidity, n (%)*	11 (11.22%)	19 (18.63%)	0.1427 ^a^
*Positivity, n (%)*	73 (72.28%)	91 (89.22%)	**0.0022 ^a^**
*Hospitalized, n (%)*	82 (83.67%)	42 (41.18%)	**<0.0001 ^a^**
*Length of stay (mean days ± SD)*	9.63 ± 8.22	5.89 ± 5.19	**0.0325 ^b^**
*Hospitalized in ICU, n (%)*	23 (23.47%)	5 (4.90%)	**0.0002 ^a^**
*Length of stay (mean days ± SD)*	14.42 ± 9.52	8.40 ± 6.22	0.8674 ^c^

^a^ Chi-square. ^b^ Student’s *t*-test. ^c^ Mann–Whitney’s U, Bold text highlights *p* value < 0.05.

## Data Availability

The data presented within this study are available upon request to the corresponding author.

## References

[1] Saunders M., Nellums L. (2022). The indirect effects of COVID-19 upon global childhood pneumonia. Public Health Pract..

[2] Hu H., Zhou T., Gao J., Ou Y., Ma A., Wang P. (2024). Economic burden and influence factors among hospitalized children with bronchiolitis or pneumonia: A multiregional study in China. Front. Public Health.

[3] Parra-Lucares A., Segura P., Rojas V., Pumarino C., Saint-Pierre G., Toro L. (2022). Emergence of SARS-CoV-2 Variants in the World: How Could This Happen?. Life.

[4] Alvis-Zakzuk N.J., Couto P., Jara J.H., Descalzo M., Rondy M., Tempia S., Vicari A. (2025). Economic Burden of Respiratory Viruses in Latin America and the Caribbean (LAC): A Scoping Literature Review. Influenza Other Respir. Viruses.

[5] Jayaraman A.S., Darekar I., Dadhich N.V., Tadepalli L.S.M., Gongwang Y., Singh S., Gavor E. (2024). Effect of the COVID-19 Pandemic on Respiratory Diseases and Their Economic Impacts. Pathogens.

[6] Mazur N.I., Caballero M.T., Nunes M.C. (2024). Severe respiratory syncytial virus infection in children: Burden, management, and emerging therapies. Lancet.

[7] Avendaño L. (2019). Infeccion respiratoria por adenovirus en pediatria: De ayer a hoy. Neumol. Pediátrica.

[8] ISP Chile Vigilancia Virus Respiratorio. https://www.ispch.cl/wp-content/uploads/2023/02/Informe-circulacion-virus-respiratorios-SE07-21-02-2023.pdf.

[9] Le-Corre N., Pérez R., Vizcaya C., Martínez-Valdebenito C., López T., Monge M., Alarcón R., Moller F., Martínez M.-T., Massardo J.-M. (2021). Relevance of codetection of respiratory viruses in the severity of acute respiratory infection in hospitalized children. Andes Pediatr..

[10] Alvarez-Moreno C.A., de Araújo E.S.A., Baumeister E., Nogales Crespo K.A., Kalergis A.M., Muñoz Medina J.E., Tsukayama P., Ugarte-Gil C. (2024). Differential Diagnosis in the Management of Acute Respiratory Infections through Point-of-Care Rapid Testing in a Post-Pandemic Scenario in Latin America: Special Focus on COVID-19, Influenza, and Respiratory Syncytial Virus. COVID.

[11] Chan W.S., Ho C.W., Chan T.C., Hung J., To M.Y., Leung S.M., Lai K.C., Wong C.Y., Leung C.P., Au C.H. (2024). Clinical Evaluation of the BIOFIRE SPOTFIRE Respiratory Panel. Viruses.

[12] Liang S.Y., Theodoro D.L., Schuur J.D., Marschall J. (2014). Infection prevention in the emergency department. Ann. Emerg. Med..

[13] Matienzo N., Youssef M.M., Comito D., Lane B., Ligon C., Morita H., Winchester A., Decker M.E., Dayan P., Shopsin B. (2021). Respiratory viruses in pediatric emergency department patients and their family members. Influenza Other Respir. Viruses.

[14] McHugh M., Tanabe P., McClelland M., Khare R.K. (2012). More patients are triaged using the Emergency Severity Index than any other triage acuity system in the United States. Acad. Emerg. Med..

[15] Acuña M., Benadof D., Yohannessen K., Leiva Y., Clement P. (2022). FilmArray^®^ Meningoencephalitis panel in the diagnosis of central nervous system infections: Stewardship and cost analysis in a paediatric hospital in Chile. BMC Pediatr..

[16] Meltzer A.C., Payette C., Heidish R., Lagunzad I., Loganathan A., Bolden T., Friedman M., Pieri M., Huang W., DeBritz D. (2025). Point-Of-Care Respiratory Diagnosis and Antibiotic Utilization in the Emergency Department: A Prospective Evaluation of Multiplex PCR. Acad. Emerg. Med..

[17] Walls T., Bowen E., Elsmore A., Lucas N. (2024). Rapid PCR Testing Reduces Antibiotic Prescribing in Children Presenting to Hospital With Lower Respiratory Tract Infections due to Influenza. Influenza Other Respir. Viruses.

[18] Vos L.M., Bruning A.H.L., Reitsma J.B., Schuurman R., Riezebos-Brilman A., Hoepelman A.I.M., Oosterheert J.J. (2019). Rapid Molecular Tests for Influenza, Respiratory Syncytial Virus, and Other Respiratory Viruses: A Systematic Review of Diagnostic Accuracy and Clinical Impact Studies. Clin. Infect. Dis..

[19] Torres J.P., Sauré D., Goic M., Thraves C., Pacheco J., Burgos J., Trigo N., Del Solar F., Neira I., Díaz G. (2025). Effectiveness and impact of nirsevimab in Chile during the first season of a national immunisation strategy against RSV (NIRSE-CL): A retrospective observational study. Lancet Infect. Dis..

[20] Zumla A., Al-Tawfiq J.A., Enne V.I., Kidd M., Drosten C., Breuer J., Muller M.A., Hui D., Maeurer M., Bates M. (2014). Rapid point of care diagnostic tests for viral and bacterial respiratory tract infections--needs, advances, and future prospects. Lancet Infect. Dis..

[21] Bhaskaran P.N., Moni M., Sathyapalan D.T.T., Jose S., Krishna A., Rose L., Sudheer A., Kishore K., Menon V. (2025). Impact of multiplex PCR respiratory viral panel testing on antibiotic utilisation in children with acute febrile and respiratory illnesses. BMJ Paediatr. Open.

